# Structural and Electrical Characterization of 2” Ammonothermal Free-Standing GaN Wafers. Progress toward Pilot Production

**DOI:** 10.3390/ma12121925

**Published:** 2019-06-14

**Authors:** Daryl Key, Edward Letts, Chuan-Wei Tsou, Mi-Hee Ji, Marzieh Bakhtiary-Noodeh, Theeradetch Detchprohm, Shyh-Chiang Shen, Russell Dupuis, Tadao Hashimoto

**Affiliations:** 1 SixPoint Materials, Inc., Buellton, CA 93427, USA; ed@spmaterials.com (E.L.); tadao@spmaterials.com (T.H.); 2 Georgia Institute of Technology, Atlanta, GA 30332, USA; chuan-wei.tsou@ece.gatech.edu (C.-W.T.); mihee.ji@gatech.edu (M.-H.J.); Bakhtiary.marzieh@gatech.edu (M.B.-N.); theeradetch.detchprohm@ece.gatech.edu (T.D.); shensc@ece.gatech.edu (S.-C.S.); dupuis@gatech.edu (R.D.)

**Keywords:** GaN, ammonothermal, bulk, substrates, NEAT, power devices

## Abstract

Free-standing gallium nitride (GaN) substrates are in high demand for power devices, laser diodes, and high-power light emitting diodes (LEDs). SixPoint Materials Inc. has begun producing 2” GaN substrates through our proprietary Near Equilibrium AmmonoThermal (NEAT) growth technology. In a single 90 day growth, eleven *c*-plane GaN boules were grown from free-standing hydride vapor phase epitaxy (HVPE) GaN substrates. The boules had an average X-ray rocking curve full width at half maximum (FWHM) of 33 ± 4 in the 002 reflection and 44 ± 6 in the 201 reflection using 0.3 mm divergence slits. The boules had an average radius of curvature of 10.16 ± 3.63 m. The quality of the boules was highly correlated to the quality of the seeds. A PIN diode grown at Georgia Tech on a NEAT GaN substrate had an ideality factor of 2.08, a high breakdown voltage of 1430 V, and Baliga’s Figure of Merit of >9.2 GW/cm^2^. These initial results demonstrate the suitability of using NEAT GaN substrates for high-quality MOCVD growth and fabrication of high-power vertical GaN switching devices.

## 1. Introduction

Gallium Nitride (GaN) semiconductor devices including light-emitting diodes (LEDs), laser diodes, and power transistors have brought about great technological change. White LEDs were invented in 1994 by adding a yellow phosphor to GaN-based blue LEDs invented the year prior. White LEDs had tremendous advantages over incumbent lighting technologies including efficiency, lifetime, color, and size. These impacts were recognized in 2014 when the Nobel Prize in Physics was awarded to the inventers of the GaN-based blue and subsequent white LED [1]. Adoption of LEDs is decreasing the energy used for lighting. In 2015, the LED-installed stock penetration of 6% in the U.S. resulted in an annual saving of 82 billion kWh. In 2035, the LED-installed stock penetration is projected to be 86% to 88%, saving 1.09 to 1.49 trillion kWh annually. That is a savings of 55%–75% compared to a scenario without solid-state lighting [2]. Since around 2006, GaN-based 405 nm blue-laser diodes have been used in Blu-ray disc players and video game consoles. The smaller wavelength of the laser compared to the 650 nm red lasers used with DVD allowed the data capacity to increase five times to 25 GB per layer on discs that are the same physical size. The increased capacity allowed feature films to be stored at a higher resolution; DVD was 720 × 480 pixels (480p) and Blu-ray is up to 3840 × 2160 pixels (4K).

While GaN-based LEDs and laser diodes are in ubiquitous consumer-facing commercial products, GaN-based power devices are in their infancy but promise a breakthrough over the incumbent silicon power devices. Power electronics are semiconductor switching devices used to control and convert electrical power flow. They are found throughout the electric grid and in electronic devices. An easily recognizable power device is the AC to DC power adapter between the wall outlet and a phone or laptop computer that converts the 110 V AC power to ~10 V DC power. Larger electronic devices typically have power electronics built into the device instead of being along the power cable. Additional examples include the electric motor drive of an electric vehicle that draws power from the battery at 350 V DC and dives the motor at three-phase 650 V AC, and a solar inverter that converts 48 V DC power to 220 V AC. Today, the overwhelming majority of power devices are made from silicon. However, silicon power device improvements are now largely limited by inherent material properties. GaN and other wide bandgap semiconductors offer higher blocking voltages, efficiency, and reliability as well as lower cooling requirements and decreased system size and weight relative to silicon devices [3]. Yole Développement, a French market research and technology analysis company, estimates that replacing silicon power devices with the wide bandgap semiconductors GaN and SiC can increase DC-AC conversion efficiency from 96% to 99%, DC-DC conversion efficiency from 85% to 90%, and AC-DC conversion efficiency from 85% to 90% [4]. There is a growing market for GaN power electronics in fast chargers, LiDAR, data centers, wireless charging, electric vehicles, and hybrid vehicles [5].

The semiconductor wafer that devices are fabricated on is called the substrate, and its material and quality affect device performance. Other crystalline substrates such as silicon, sapphire, germanium, gallium arsenide, and indium phosphide are available at low cost. That is not the case for GaN. GaN substrates are notoriously difficult to produce and, therefore, are expensive. To circumvent this issue, most GaN devices, especially LEDs, are fabricated on a non-native substrate such as sapphire or silicon that has a thin film of GaN grown on the surface. However, because the lattice constant and the coefficient of thermal expansion of the non-native substrate are not the same as GaN, the thin GaN film is stressed, strained, and bowed, resulting in many defects where the atoms of the crystal lattice are misaligned. GaN on Si typically has 10^9^ dislocations per square centimeter. These defects, called edge, screw, and mixed dislocations are known to degrade the performance of semiconductor devices [6]. For example, during the development of GaN laser diodes at Sumitomo, device lifetimes were shown to increase approximately linearly as dislocation density went down [7]. To meet the high standards of commercial products, laser diodes had to be grown exclusively on GaN substrates with a dislocation density on the order of 10^6^ dislocations per square centimeter. For power devices, dislocation density can affect breakdown voltage, leakage current, reliability, and operating life [8]. For example, Usami et al. recently identified nanopipes formed from the coalescence of edge and mixed dislocations during epitaxial growth as leakage paths in vertical PN diodes. They accomplished this by superimposing emission microscope data with etch pit data and investigating the pits that coincided with the leakage spots using transmission electron microscopy [9]. In addition to having a low dislocation density, growing GaN devices on GaN substrates (GaN-on-GaN) allows vertical device architectures which have better thermal management, higher reliability, and high breakdown voltages using small devices as compared to lateral devices [10]. Recent research on high-power vertical devices on bulk GaN can be found in references [11,12,13,14,15,16]. It has also been shown that GaN-on-GaN LEDs can operate efficiently at high current density unlike heteroepitaxial LEDs which experience a strong efficiency droop as current density is increased [17].

Most GaN substrates available today are grown one at a time by hydride vapor phase epitaxy (HVPE). A thick, 0.5–2 mm, GaN film is grown on a foreign substrate, usually sapphire or gallium arsenide, over the course of about 5–20 h. The quasi-bulk GaN is then made free-standing by removing the substrate by grinding, chemical etching, laser liftoff, or use of a mask that causes separation on cool down. HVPE grown GaN typically has 10^6^ to 10^7^ dislocations per square centimeter. Dislocation reduction in HVPE GaN is difficult because it is grown on a foreign substrate and faces technical difficulties with growing thicker crystals such as parasitic nucleation, pit formation, and clogging of the reactor exhaust from ammonium chloride precipitation.

The best quality GaN substrates reported in the literature were grown by the ammonothermal method [18]. Ammonothermal growth is a solution growth technique that produces true-bulk GaN. It can potentially address both issues holding back GaN substrates, quality and cost. In the ammonothermal method, high-temperature (>400 °C) high-pressure (>100 MPa) supercritical ammonia inside an autoclave dissolves polycrystalline GaN from the nutrient region and recrystallizes it on single-crystal GaN seeds in the growth region, resulting in bulk GaN boules. Unlike HVPE, many substrates are produced from a single run. In ammonothermal growth, increasing the number of substrates produced per run provides a clear path to cost reduction because many of the production costs such as labor, ammonia, mineralizer, gaskets, and electricity are fixed. Because ammonothermal GaN boules are grown on GaN seed crystals, there is not a large lattice mismatch that strains the crystal and causes defects. This means that the crystal quality of the seed is maintained or improved in the boule. Ammonothermal GaN substrates typically have 10–100 times less defects per unit area than HVPE GaN. Each GaN boule is sliced in a wire saw yielding multiple substrates.

In the GaN semiconductor system, the biggest bottleneck is the availability of native substrates that are both low-cost and low-defect. The aim of this work is to bring low-defect true-bulk GaN substrates to market with a clear path to lowering cost using our proprietary near equilibrium ammonothermal (NEAT) method. The NEAT method is optimized to enable excellent quality growth for several months at a steady growth rate, allowing each seed to be grown thick enough so that it can be sliced into several wafers. This is achieved by tuning the temperature gradient in the reactor to minimize parasitic nucleation. If parasitic nucleation occurs, the growth rate on the seeds decreases over time because an increasing fraction of the dissolved GaN is diverted to growing crystals on the parasitic nuclei rather than the seeds. Growing close to the equilibrium is also important for maintaining or improving crystal quality. When growing faster, we found that it was more likely that the grown crystal would be lower quality than the seed as measured by the full width at half maximum (FWHM) of the X-ray rocking curve. 

## 2. Materials and Methods

Single-crystal *c*-plane GaN boules were grown using SixPoint’s proprietary NEAT method which has been published previously [19,20,21,22]. Growth took place over 90 days in a Ni-Cr-based superalloy autoclave using 99.99994% purity supercritical ammonia as a solvent. A growth temperature within the range of 450–600 °C was established using an array of furnaces. Polycrystalline GaN nutrient dissolved and was transported to the seed region by convection and/or diffusion. Commercial HVPE-grown *c*-plane free-standing GaN wafers were used as seeds. The seeds were screened by X-ray diffraction (XRD) mapping, where the uniformity of FHWM values across the seed must be below a threshold to ensure good quality, crack-free growth [19]. An ammonia fill factor of 40%–60% was used, resulting in a pressure in the range of 100–300 MPa. Solubility of GaN was increased by the addition of a mineralizer.

After growth, the boules underwent characterization. Thickness was measured using a Mitutoyo digital micrometer, weight was measured using a Cole-Parmer Symmetry analytical balance, and photographs were taken using a Canon Powershot SX510 HS camera and a Canon 9000F Mark II scanner using the backlight. Surface morphology was observed using a Nomarski microscope (Nikon AZ100, Tokyo, Japan) and captured using a Lumenera Infinity2 microscopy camera.

Structural quality and uniformity was assessed by measuring the FWHM of X-ray rocking curves in the 002 and 201 reflections. X-ray diffraction measurements were done using a Philips PANalytical high-resolution four-axis X-ray diffractometer. Cu Kα X-ray radiation from a Cu anode operated at 40 mA and 40 kV passed through a four-crystal Ge (002) monochromator and two perpendicular 0.3 mm divergence slits resulting in a beam spot of 0.3 mm × 1.0 mm for the 002 reflection and 1.17 mm × 0.52 mm for the 201 reflection. The system was operated in point focus mode and the detector slits were open. Four lines of XRD measurements were performed across each seed, growth, and wafer. Across each line, the measurement was repeated every 0.6 to 0.9 mm. Before a line of measurements began across the wafer at a given Y value, an alignment scan was done at X = 0, and the ω angle value there was set to 17.2833°, the theoretical value. This was done so that when a comparison graph of ω angle as a function of position on the substrate was made, the lines crossed at X = 0 mm and ω peak position = 17.2833°. In addition to calculating the radius of curvature from adjacent measurements, which we refer to as the local curvature, we also calculated what we call the global curvature. The global curvature was calculated using the ω peak positions from all the repeated 002 scans done every 0.9 mm across the sample at a given y position. When the change in ω peak positions are small enough, the formula for radius of curvature is

(1) $$R_{C}=\frac{x_{1}-x_{2}}{\omega_{1}-\omega_{2}}$$

where $\omega_{1}$ is the omega peak position measured at $x_{1}$, and $\omega_{2}$ is the omega peak position measured at $x_{2}$ [23]. In a plot of ω peak angle as a function of x position, this formula is the inverse slope of a line. So to calculate the global curvature, we plotted the ω peak angle from each diffractogram as a function of its x position on the wafer, fitted a line to the data using a least squares fit, took the inverse of the slope, and converted the units to meters. The graph can be illuminating if the curvature is not constant across the wafer or for example there is a grain boundary causing a discontinuity.

After characterization, boules were processed into wafers. Rounding was done, miscut alignment was performed, and slicing was done using a Takatori wire saw. Wafers were ground to refine the miscut and to planarize them. Surface and subsurface damage was removed by polishing and lapping with diamond slurry, and chemical mechanical polishing (CMP) with colloidal silica. Subsurface damage was monitored using a glancing angle X-ray measurement technique detailed in Letts et al. [24]. The full width at 5000th max (FW5000M) of the ω 114 reflection peak is measured at five locations on the wafer and compared to the diffractogram of an undamaged as-grown GaN crystal surface. Atomic force microscopy (AFM) was done to confirm the CMP produced an atomically smooth surface with a step structure due to the intentional miscut angle.

Dislocation density was estimated using a defect-selective wet etching method from Zhuang et al. [25]. The method is able to decorate defects because the etch rate at defects is greater than the etch rate in defect-free areas. Samples were etched in a eutectic alloy of 51.5 mol% NaOH and 48.5 mol% KOH at 230 °C for 30 s. After etching, the samples were observed using a Nomarski microscope and images were taken at nine locations. A 100 × 100 µm square was cropped from each image and the number of etch pits was counted visually.

**Figure 1:**
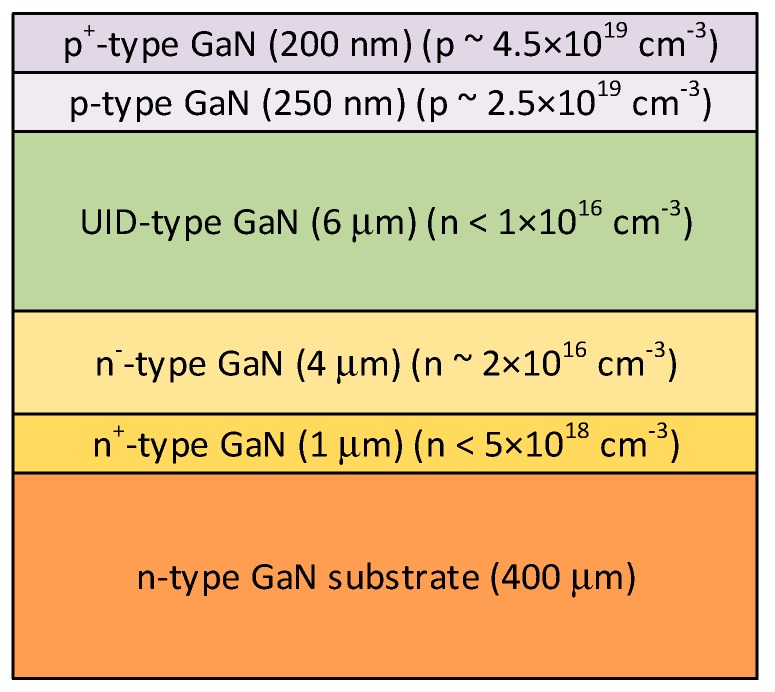
The epitaxial layer structure of the PIN rectifier grown at Georgia Tech on a NEAT GaN substrate.

Vertical GaN PIN rectifiers were fabricated on a NEAT GaN substrate at Georgia Tech using a Schottky field plate in combination with a nitrogen-ion-implanted device isolation. The epitaxial layers were grown by metalorganic chemical vapor deposition (MOCVD) in an AIXTRON 6 × 2 CCS reactor. The layers are shown in Figure 1 and consist of a 1-µm *n*^+^-GaN layer (*n* = 5 × 10^18^ cm^−3^), a 4-µm lightly doped *n*^-^-GaN drift layer (n ~ 2 × 10^16^ cm^−^^3^), a 6-µm unintentionally doped (UID) drift layer (residual free electron concentration [n] < 1 × 10^16^ cm^−^^3^), a 250-nm *p*-GaN layer ([Mg] ~ 2.5 × 10^19^ cm^−^^3^), and a 200-nm higher doped *p*^+^-GaN layer ([Mg] ~ 4.5 × 10^19^ cm^−^^3^). The device fabrication started with a back-side *n*-type Ti/Al-based contact formation, followed by a Ni/Ag-based *p*-type ohmic contact. The ohmic contact (Ni/Ag/Ni/Au = 2.5/50/30/50 nm) on *p*-type GaN was deposited, followed by rapid thermal annealing at 475 °C for 75 s in dry air ambient (20% O_2_ and 80% N_2_). A nitrogen ion (N^+^) implantation was employed for inter-device isolation. A Schottky field plate was also included for device-edge field termination. The device passivation was done using a 7-nm-thick ALD-grown Al_2_O_3_ layer. The high-voltage characteristics were measured using a curve tracer (Tektronix 370B, Beaverton, Oregon, USA), which shows a voltage limit when the impedance drops.

## 3. Results

**Figure 2:**
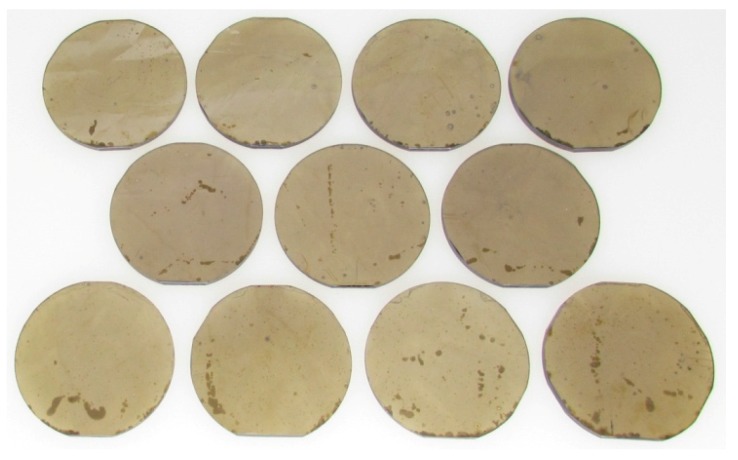
Eleven rounded 2” *c*-plane GaN boules grown by the NEAT method in a single run.

We grew 11 single-crystal *c*-plane GaN boules. Figure 2 shows an image of the boules after rounding. XRD mapping revealed the boules had an average 002 FWHM of 33 ± 4 arcsec and an average 201 FWHM of 44 ± 6 arcsec. The crystal quality of the seeds was a strong predictor of crystal quality in the boules. The correlation coefficient (r) between the average FWHM of the seed and the boule was 0.95 and 0.81 for the 002 and 201 reflections respectively. The average global radius of curvature of the boules was 10.16 ± 3.63 m. The radius of curvature of the seed and the boule were highly negatively correlated with r=−0.96. The correlation is negative because the lattice curvature flips during growth. This occurs due to different impurity incorporation, largely oxygen, in the HVPE versus ammonothermal growth environments. The oxygen concentration in the crystal was 2.2 × 10^19^ atoms per cm^3^ as measured by secondary ion mass spectrometry (SIMS) at EAG Laboratories. The visible imperfections on the crystal surface in Figure 2 result from the cool down at the end of the run when the mineralizer precipitates out of solution. These lightly etched areas on the surface are inconsequential and removed after processing the boules into wafers.

**Figure 3:**
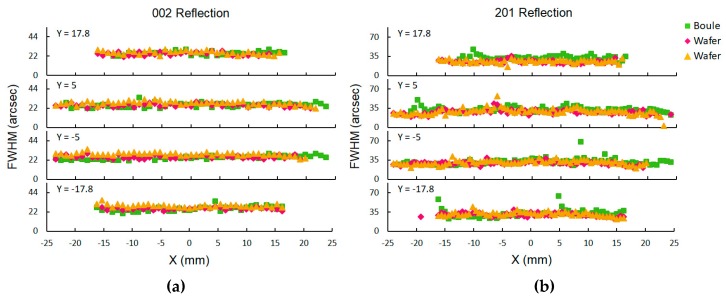
X-ray diffraction mapping of the 002 reflection FWHM (**a**) and the 201 reflection FWHM (**b**).

XRD mapping of a boule and two wafers sliced from it are shown in Figure 3. The 002 reflection FWHM values were 25 ± 2, 25 ± 2, and 28 ± 2 arcsec for the boule and wafers 1 and 2 respectively. This result is excellent in both absolute terms and in uniformity. The 201 reflection FWHM values, shown in Figure 3b, were 32 ± 4, 28 ± 4, and 29 ± 5 arcsec for the boule and wafers 1 and 2 respectively. Again, this shows good uniform crystal quality across each wafer and the boule they were sliced from. Figure 3 also demonstrates the similarity of the wafers to the boule they were sliced from.

**Figure 4:**
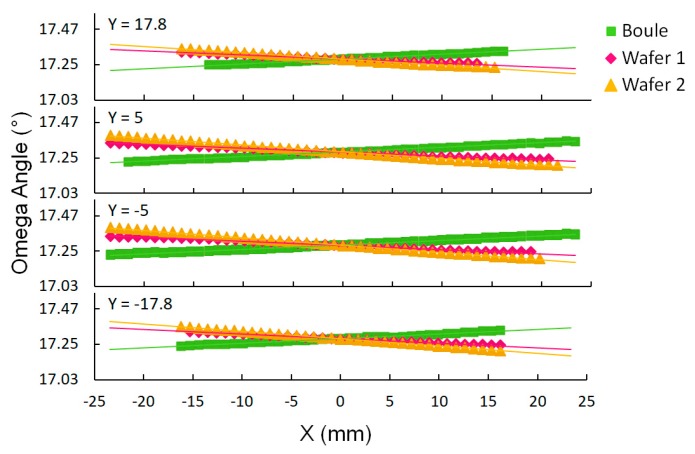
002 reflection ω peak angle values plotted as a function of position on the wafer. Global curvature is calculated from the slope of each line. The more horizontal the line is, the flatter the crystal lattice. The boule was measured on the nitrogen polar face, and the wafers were measured on the gallium polar face.

The radius of curvature ($R_{C}$) is calculated by looking at the difference in ω peak positions measured at two X positions on the crystal. Formula 1 is recognized as the inverse slope of the lines in Figure 4. We call the radius of curvature calculated from two points the local curvature, and the radius of curvature calculated from the many points across the entire sample the global curvature. This graphical representation makes analysis intuitive; the more horizontal the line, the flatter the crystal lattice. The average global radius of curvature was 19.95 ± 0.49, −22.10 ± 1.17, and −13.37 ± 1.06 m based on the four lines for the boule, wafer 1, and wafer 2 respectively. This shows the degree to which the crystal lattice is flat and the stress on the lattice is low. The curvature of the boule has the opposite sign as the wafers because the boule was measured on the nitrogen polar face and the wafers were measured on the gallium polar face. The average global curvature for all 11 boules was 10.16 ± 3.63 m.

**Figure 5:**
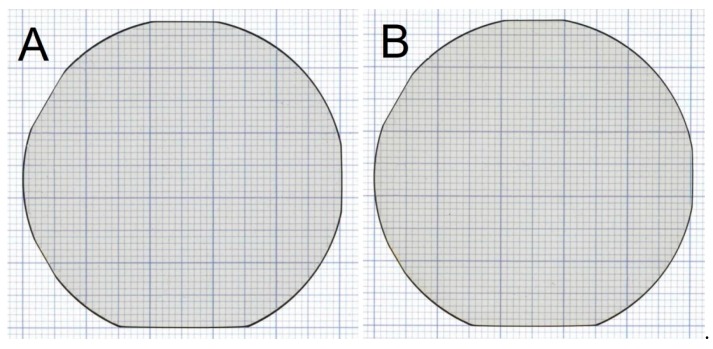
Backlit scanner images of the N side of two CMPed NEAT GaN wafers (**A**,**B**) from the same boule.

Backlit scanner images of the N side of two CMPed NEAT wafers from the same boule are shown in Figure 5. The wafers are rounded and some unintended minor flats remain due to the way the crystal was grown. These additional flats were due to a technical problem that has been addressed and are not expected in future growth. The wafers have a subtle amber color due to oxygen impurity incorporation. The oxygen concentration was 2.2 × 10^19^ atoms per cm^3^ making the substrates *n*-type. Wafer thickness falls into the target range of 350–400 µm. 

**Figure 6:**
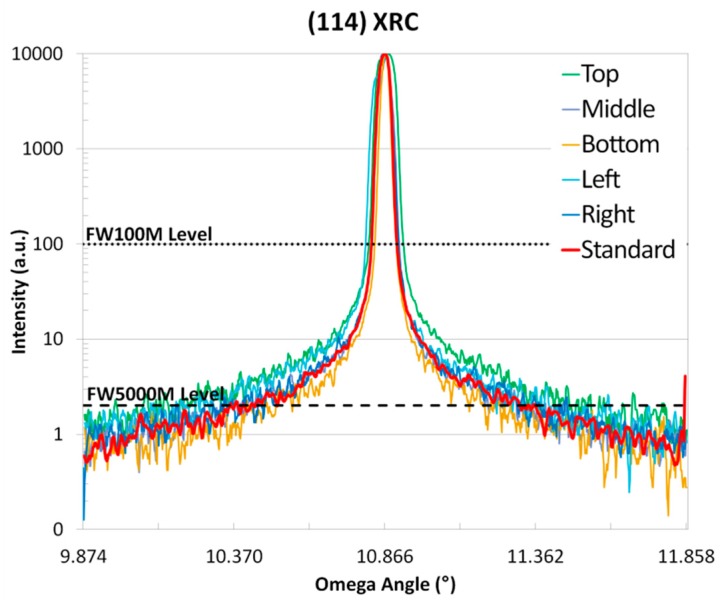
Omega 114 X-ray rocking curves measured at five locations on the G-polar side of a *c*-plane CMPed NEAT GaN wafer. A diffractogram of an as-grown NEAT GaN boule (shown in red) is used as a standard since its surface is free of any damage caused by surface processing. The width of the peaks at the 5000M level is used to quantify surface damage. The data has been smoothed with a smoothing ratio of 1/5 for easier visual comparison, and the intensity has been normalized to 10,000 counts, an arbitrary unit.

Glancing angle XRD measurements of the 114 reflection were performed to measure the quality and uniformity of the surface finish after CMP. The presence of remnant surface and subsurface damage from surface processing causes increased scattering intensity in the tails of the ω peak in the 114 reflection. The ω peak from the 114 reflection was measured at five locations on the wafer and compared to the diffractogram of an as-grown crystal surface as shown in Figure 6. A diffractogram of an as-grown NEAT GaN boule is used as a standard since its surface is free of any damage caused by surface processing. The diffractograms were smoothed with a smoothing ratio of 1/5 for easier visual comparison, and the intensity was normalized to 10,000 counts, an arbitrary unit. The intensity axis is plotted on a log scale to emphasize the scattering in the tails of the peak where the surface damage is quantified by measuring the full width of the peak at one-five-thousandth of the maximum intensity (FW5000M). This wafer displays good quality and uniformity of CMP as shown by the similarity of the five diffractograms to each other and to the diffractogram of the undamaged boule. The average FW5000M was 3690 ± 1160 arcsec, 11% larger than the as-grown crystal surface. The surface finish was also checked by AFM and showed a clear atomic step structure.

Defect selective etching was carried out on a wafer. The number of etch pits counted in a 100 µm square varied from 31 to 80 across the nine locations measured. The average dislocation density was 3.0 ± 0.9 × 10^5^ cm^−2^. This is in line with dislocation density measurements on crystals from previous growths measured more accurately with synchrotron topography which showed dislocation densities in the range of 1–2 × 10^5^ cm^−2^. Defect selective etching is carried out sparingly because it is destructive.

**Figure 7:**
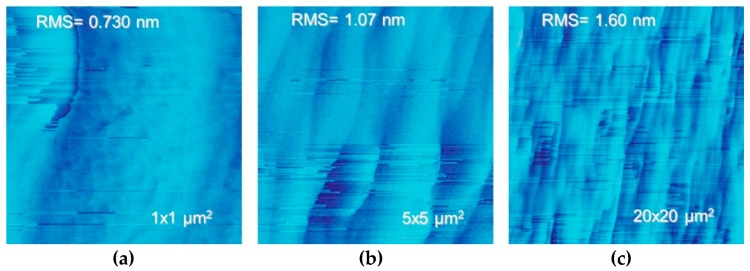
AFM images after epitaxial growth of the PIN device layers (**a**,**b**,**c**). The horizontal stripes are artifacts.

To demonstrate the usability of our substrates for high power devices, we partnered with three universities: Cornell, Georgia Institute of Technology, and Virginia Institute of Technology under the Advanced Research Projects Agency-Energy (ARPA-E) Strategies for Wide-Bandgap, Inexpensive Transistors for Controlling High-Efficiency Systems (SWITCHES) program. Each university successfully grew either high power PN or PIN diodes on our substrates using MOCVD. AFM images after epitaxial growth of the PIN device layers from Figure 1 are shown in Figure 7. The RMS roughness of the surface after epitaxial growth was 0.73, 1.07, and 1.60 nm over a 1 × 1, 5 × 5, and 20 × 20 µm square respectively. Note the horizontal stripes are artifacts.

**Figure 8:**
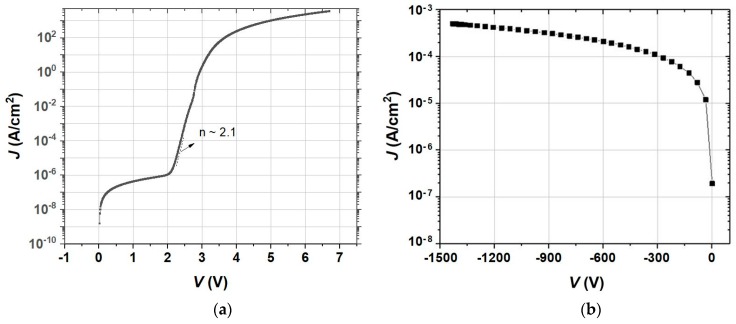
Forward- (**a**) and reverse- (**b**) biased *I-**V*** characteristics of a 120-µm diameter PIN diode grown on SixPoint’s NEAT GaN substrate.

The forward and reverse biased electrical characteristics for a 120 µm diameter PIN diode grown at Georgia Tech are shown in Figure 8. The forward- and reverse-biased electrical characteristics for a 120-µm diameter PIN diode grown at Georgia Tech. The RMS roughness of the surface after epi growth was 0.73, 1.07, and 1.60 nm over a 1 × 1, 5 × 5, and 20 × 20 µm square respectively. The forward-biased characteristics showed a turn-on voltage of 3.7 V at a current density (J) of 100 A/cm^2^. The ideality factor of the diode was 2.08 based on forward *I-V* results in the voltage range of 2.3 to 2.5 V, exhibiting typical space-charge-recombination-dominant diode characteristics for GaN PIN rectifiers. The specific on-resistance (*R*_ON_*A*) was 0.4 mΩ·cm^2^ at J = 3.5 kA/cm^2^. By excluding the probe resistance using a Kelvin measurement approach, Georgia Tech also confirmed that *R*_ON_*A* has a value of 0.22 mΩ·cm^2^ at *V* = 7 V. In reverse-bias, this device exhibits a blocking voltage of 1430 V with J < 5 × 10^-4^ A/cm^2^. Breakdown occurred at the edge of the device, indicating the fabrication process is not yet mature enough to perform a one-to-one comparison of NEAT GaN substrates to other substrates. It is not clear whether this was a hard breakdown. The built-in potential was determined to be ~4 V from the *C-V* results. The corresponding Baliga’s Figure of Merit is >9.2 GW/cm^2^. The high blocking voltage performance in combination with low on-state resistance characteristics demonstrated the suitability of the NEAT GaN substrate to facilitate high-quality MOCVD growth and fabrication of high-power vertical GaN switching devices.

## 4. Discussion

The crystal quality of the GaN grown in this run is better than or in line with the best HVPE GaN substrates available. HVPE GaN substrates are available from Sumitomo Electric, Sciocs, Saint Gobain (Lumilog), and Mitsubishi Chemical with dislocation densities of 10^6^, 10^6^, ≤10^7^, and likely 10^6^ respectively [23,26,27,28]. However, the quality of the GaN grown in our pilot production autoclave still lags behind the GaN grown in our small research reactors. We are confident that with further optimization we will make further improvements in crystal quality. The initial seed has a large effect on the structural quality after growth. We previously investigated this using seeds from different manufacturers [19]. We learned that having a uniform grain structure in the seed was critical for preventing crack formation. To evaluate the uniformity of the grain structure, we look at the standard deviation of the 201 FWHM from an XRD map like that shown in Figure 3. We have found that seeds with a 201 FWHM standard deviation below a certain threshold will improve after growth in optimized growth conditions [19]. Additionally, FWHM values improve more for seeds with large FWHM values than for seeds with good FWHM values. The slowdown in improvement is in agreement with the theory presented by Mathis et al. [29]. One way to further improve crystal quality may be to re-grow on our own material. This could be done by placing an as-grown boule or a processed NEAT GaN substrate back in the autoclave for a second growth. This area of interest is under investigation.

Another path towards further improvements in crystal quality that warrants attention is to continue reducing the oxygen concentration in the ammonothermal environment. The oxygen is high in ammonothermal growth because the growth reactor is a closed system and cannot be continuously purged. Any oxygen inside the reactor is dissolved into the solution and can incorporate into the GaN as it grows. The supercritical ammonia solvent strips the adsorbed oxygen from everything in the reactor including the nutrient, mineralizer, and all the internal metal surfaces such as the autoclave walls into solution. Pimputkar et al. theorizes that the high level of oxygen, which is an electron donor, increases the free electron concentration, resulting in a higher sub-bandgap absorption coefficient [30]. This sub-bandgap absorption is in the visible range, causing the crystals to be colored. Their theory is in agreement with our improvements in the coloration and absorption coefficient observed with a reduction in oxygen concentration. Likewise, intentionally doping with oxygen degraded the coloration and absorption coefficient [20]. Since LED makers want substrates that do not absorb light, reducing the absorbance coefficient of NEAT GaN could open up a new market for NEAT GaN in ultra-high-brightness LEDs. High electrical conductivity of the substrate is good for vertical power devices. We confirmed with SIMS analysis that the high level of oxygen in the substrate does not diffuse into the epi layers. So, oxygen atoms in the substrate do not adversely affect the power device. Another issue that arises due to oxygen incorporation is the difference in the lattice constant between GaN grown by HVPE and our ammonothermal method. Although the difference is small and does not cause much defect formation, it is enough to cause the lattice curvature to flip. Keeping the lattice spacing constant throughout the seed and growth would decrease strain and the probability of crack formation.

Initial results from device makers are encouraging. All groups successfully grew epitaxial device layers. This was good feedback that showed our backend processing including miscut control and CMP are acceptable. Working closely with device-makers helped instruct us on the critical properties of the substrates that epi growers and device manufactures care about including miscut angle, CMP, substrate thickness, physical bow, lattice curvature, and consistent color. Achieving an ideality factor of 2.08, a high breakdown voltage of 1430 V, and Baliga’s Figure of Merit of >9.2 GW/cm^2^ was a good demonstration of the NEAT GaN wafer’s potential.

Results presented here are beginning to fulfill the promise of the ammonothermal method to scale up GaN substrate production. The 11 boules grown in this run represent about 11% of the capacity of the reactor. To reach pilot production SixPoint must increase the number of seeds. In another experiment using lower quality seeds the reactor was operated at over 80% capacity and the weight gain per seed crystal only decreased by 7%. This demonstrated that our current growth condition is suitable for much higher volume production. SixPoint will continue to increase the number of boules grown in each run. The number of wafers sliced from each boule is expected to increase as well. Miscut alignment before slicing has improved, reducing the required thickness allocated to refining the miscut after slicing, allowing for a decrease in the slicing thickness and an additional wafer per boule. 

## 5. Conclusions

Ammonothermal GaN growth at SixPoint Materials has made substantial progress towards scaling up the NEAT method in a pilot production autoclave. Eleven *c*-plane GaN boules were grown from free-standing HVPE GaN substrates in a single 90 day growth period. The grown boules had excellent crystal quality with an average FWHM of the X-ray rocking curves of 33 ± 4 in the 002 reflection and 44 ± 6 in the 201 reflection using 0.3 mm divergence slits. The boules had an average radius of curvature of 10.16 ± 3.63 m. These 11 boules were approximately 11% of the capacity of the reactor. In an experiment using low-quality GaN seeds, SixPoint tested increasing the number of seeds to 80% capacity and the weight gain per seed only decreased 7% indicating the current growth condition is suitable for higher volume production. SixPoint will scale up the number of good seeds per growth as fast as the budget allows.

Boules were rounded, sliced into wafers, ground for miscut alignment and planarization, lapped, polished, and CMPed. CMP quality was evaluated using a glancing angle X-ray diffraction technique which revealed that the scattering from our finished substrates was comparable to the surface of an as-grown GaN boule that was free from surface processing-related damage. Additionally, CMP quality was validated by the fact that our partners at Virginia Tech and Georgia Tech both successfully grew good epitaxial layers by MOCVD. Finally, devices grown on our substrates exhibited good performance. A 120-µm diameter PIN diode grown by Georgia Tech had an ideality factor of 2.08, a high breakdown voltage of 1430 V, and Baliga’s Figure of Merit of >9.2 GW/cm^2^.
